# Supplementary material for: The Comprehensive Approach to Preparation and Investigation of the Eu3+ Doped Hydroxyapatite/poly(L-lactide) Nanocomposites: Promising Materials for Theranostics Application
Source: Nanomaterials (Basel). 2019 Aug 10;9(8):1146. doi: 10.3390/nano9081146 (PMC6724068; doi:10.3390/nano9081146)
Supplement: Supplementary file 1 [file nanomaterials-09-01146-s001.pdf]

nanomaterials

Supplementary information

The Comprehensive Approach to Preparation and Investigation of the Eu^{3+} Doped Hydroxyapatite/poly(L-lactide) Nanocomposites: Promising Materials for Theranostics Application

Katarzyna Szyszka [1], Sara Targonska [1], Malgorzata Gazinska [2], Konrad Szustakiewicz [2] and Rafal J. Wiglusz [1,3,*]

[1] Institute of Low Temperature and Structure Research, Polish Academy of Science, ul. Okolna 2, 50-422 Wroclaw, Poland
[2] Wroclaw University of Science and Technology, Polymer Engineering and Technology Division, Wyb. Wyspianskiego 27, 50-370 Wroclaw, Poland
[3] Centre for Advanced Materials and Smart Structures, Polish Academy of Sciences, Okolna 2, 50-950 Wroclaw, Poland
* Correspondence: r.wiglusz@intibs.pl; Tel.: +48-071-3954-159

Received: 3 July 2019; Accepted: 6 August 2019; Published: date

S3. Results

S3.1. Morphology

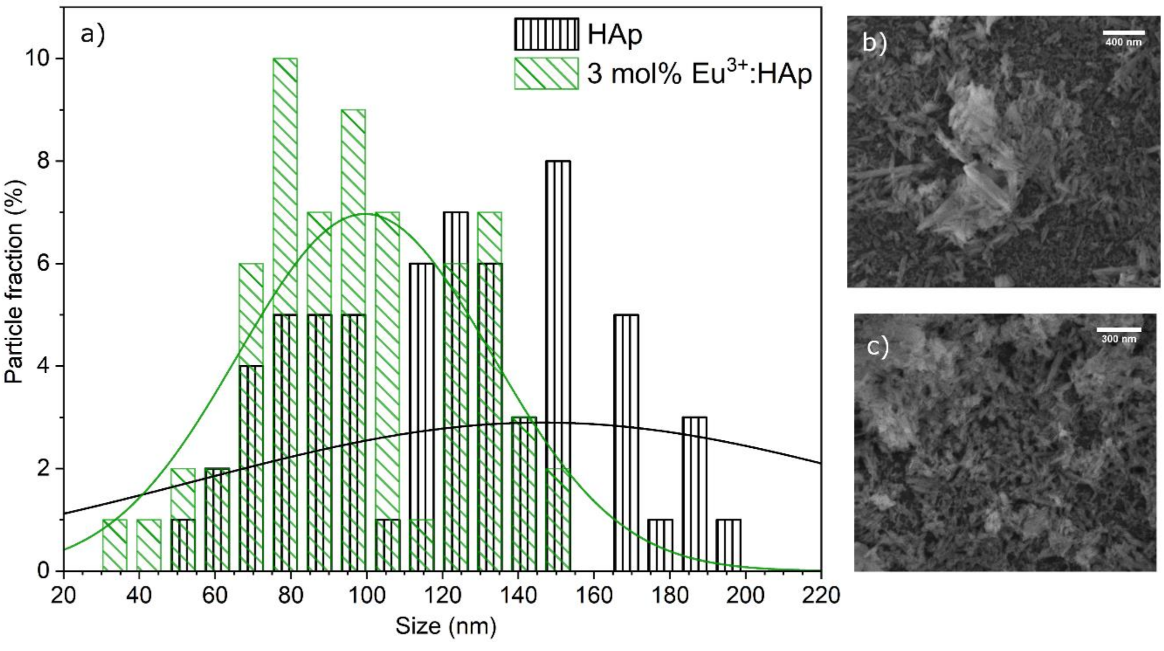

Figure S1. Particle size distribution based on SEM images (a), SEM image of HAp (b) and 3 mol% Eu^{3+}:HAp particles (c).

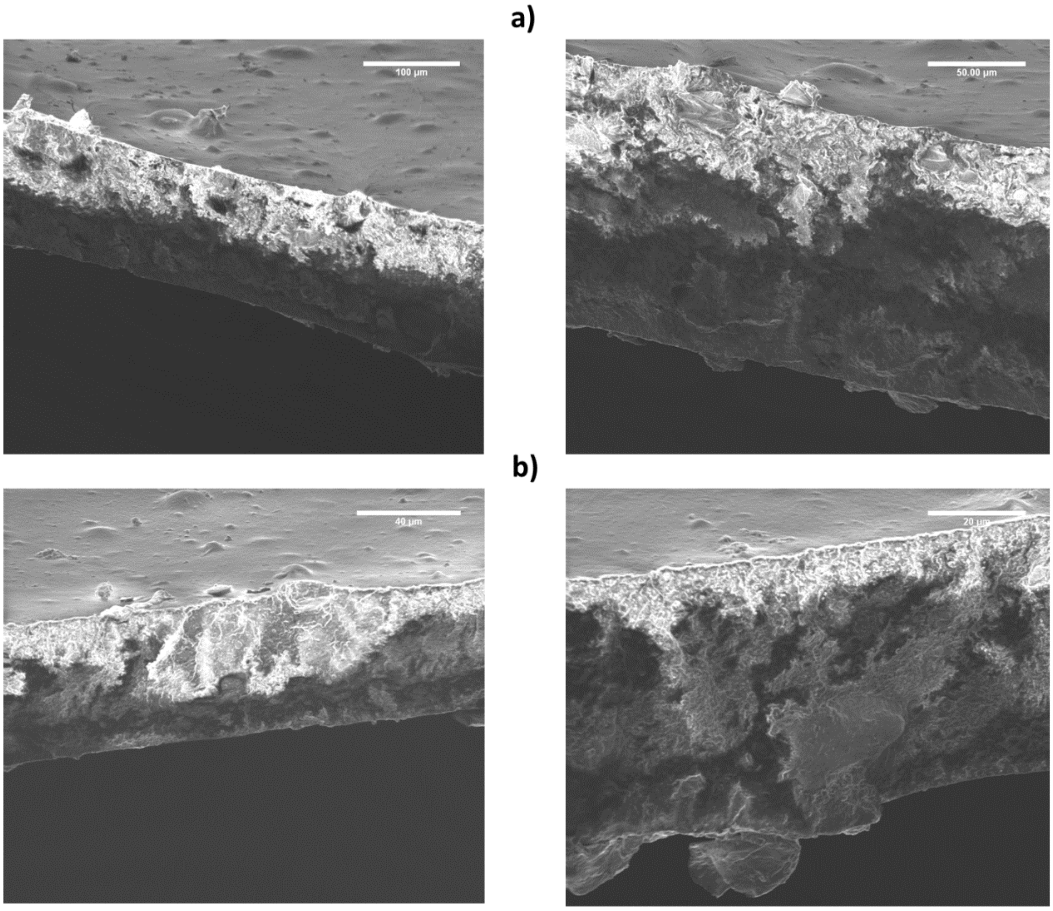

Figure S2. SEM images of breakthroughs for PLLA/HAp (a) and PLLA/5 mol% Eu^{3+}:HAp (b).

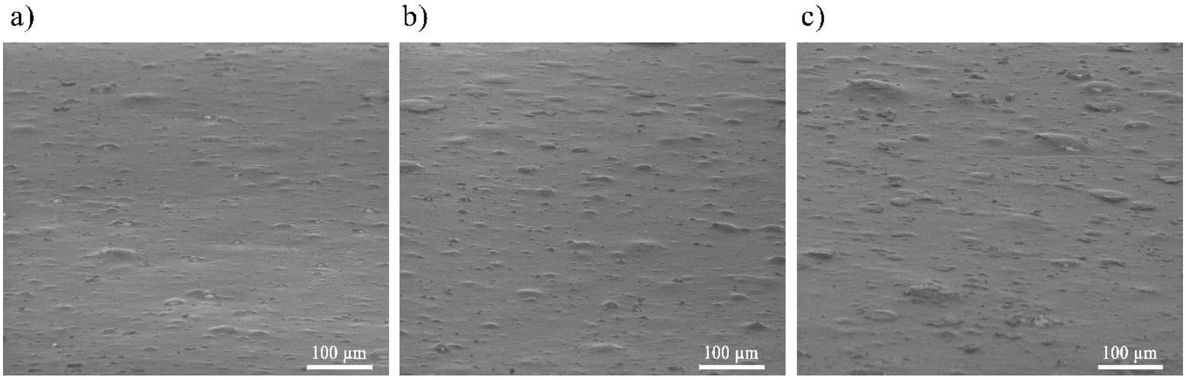

Figure S3. SEM images of composite surface for PLLA/1 mol% Eu^{3+}:HAp (a), PLLA/3 mol% Eu^{3+}:HAp (b), PLLA/5 mol% Eu^{3+}:HAp (c).

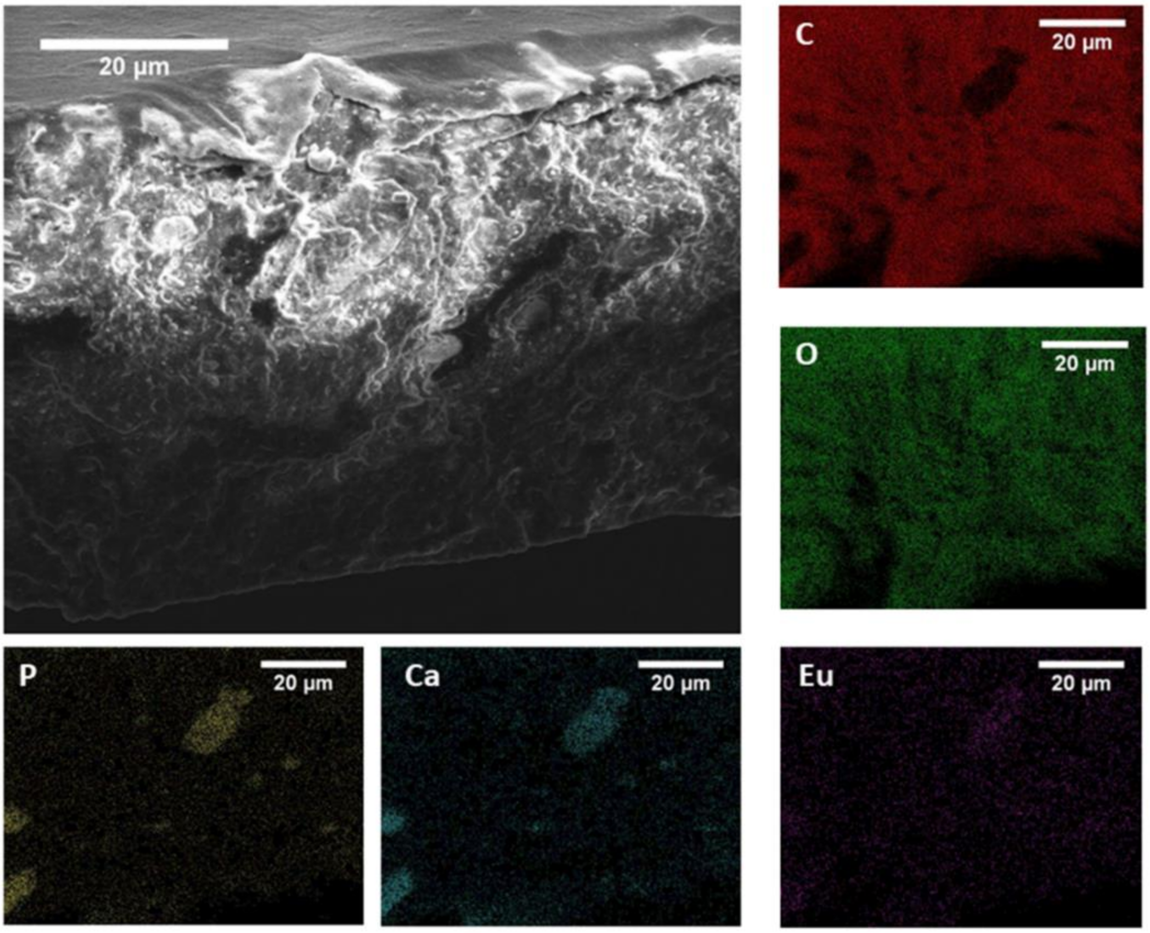

Figure S4. SEM-EDS element maps of the PLLA/5 mol% Eu^{3+}:HAp composites.

S3.2. Structural analysis of Eu^{3+} doped composites

The crystal phase purity of $Ca_{10}(PO_4)_6(OH)_2$ nanocrystals doped with Eu^{3+} ions were checked by the powder XRD technique and compared with the reference standard of the $Ca_{10}(PO_4)_6(OH)_2$ host lattice (ICSD – 180315 [1]). All synthesised nanoapatites were characterised by detectable crystallinity at entire range of proposed dopant concentration (**Figure S5**). Moreover, no extra diffraction peaks corresponding to any impurities were detected, which means that the single crystalline phase of hydroxyapatites in entire range of proposed dopant concentration of optically active ions were obtained. Based on the recorded diffraction patterns, it is possible to affirm that Eu^{3+} ions were successfully incorporated into the host lattice by occupying the Ca^{2+} ions' sites as well as that incorporating of Eu^{3+} ions with different concentrations does not have destabilize impact on the hydroxyapatite crystal structure. It means that Eu^{3+} ions are well-dissolved in the host lattice.

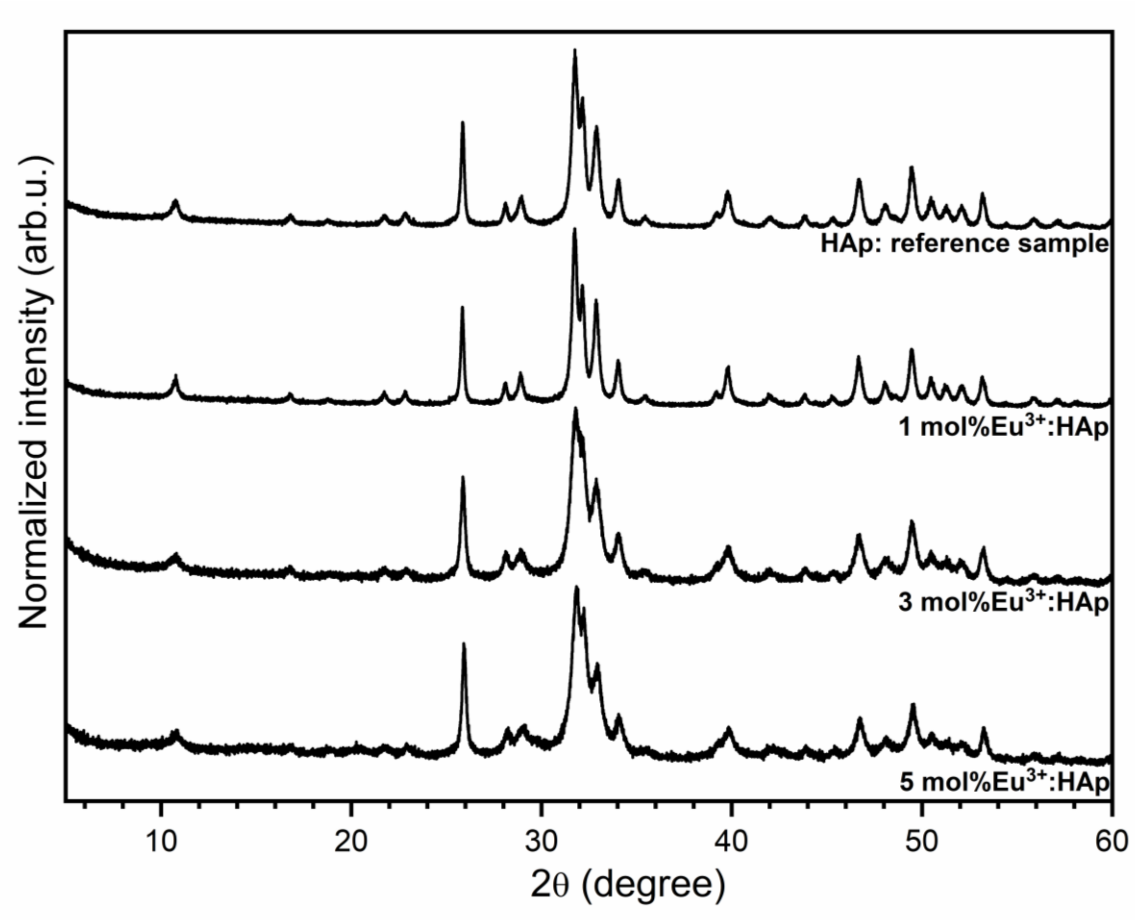

Figure S5. X-ray powder diffraction patterns of x mol% Eu^{3+}:$Ca_{10}(PO_4)_6(OH)_2$ (where x = 0 – 5).

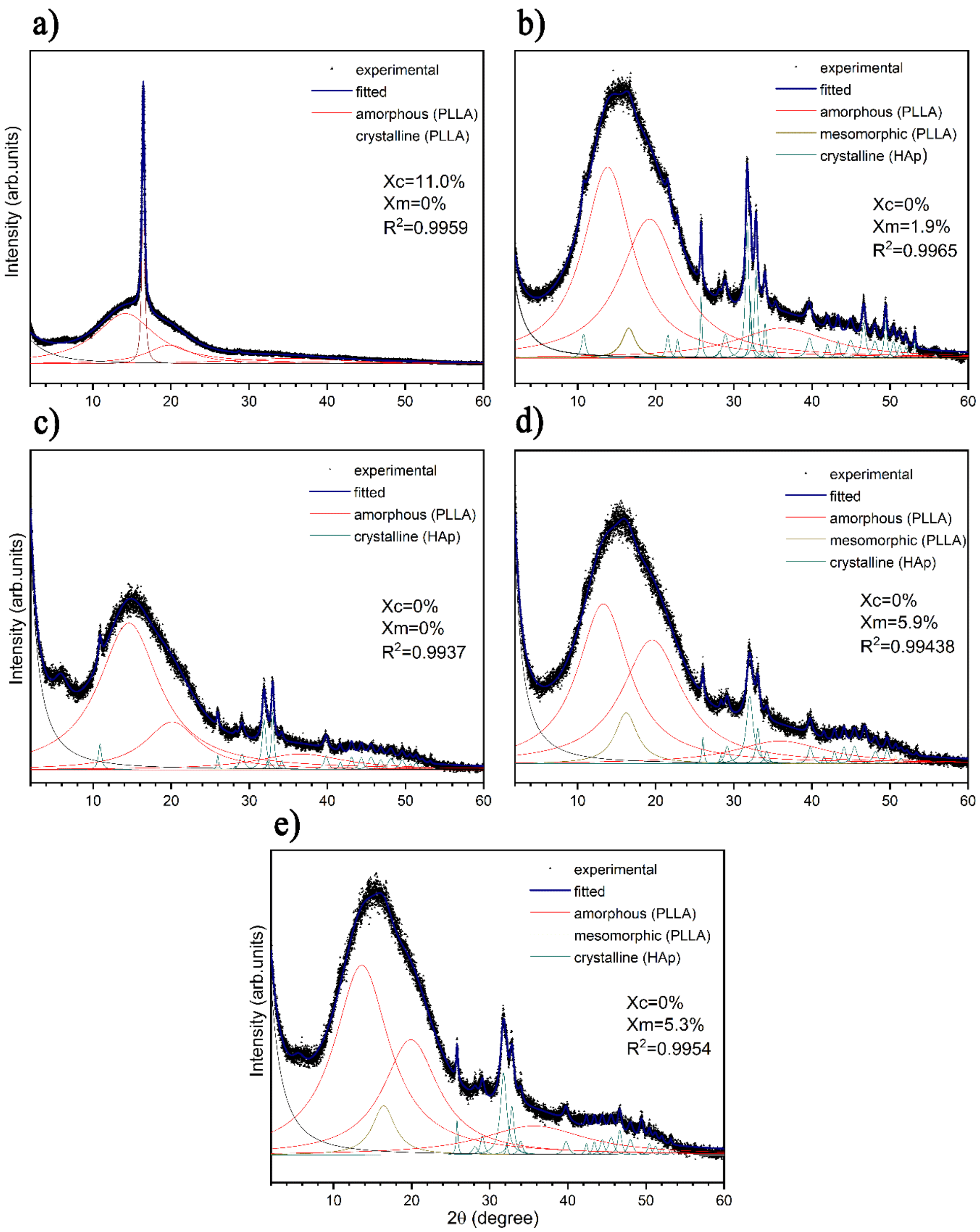

Figure S6. Deconvolution of WAXS patterns of the unmodified PLLA (a); the PLLA/HAp (b); the PLLA/1 mol% Eu^{3+}:HAp (c); the PLLA/3 mol% Eu^{3+}:HAp (d) and the PLLA/5 mol% Eu^{3+}:HAp (e).

The IR spectra of HAp consist of typical active vibrational bands of phosphate groups (PO_4^{3-}) at 472.2 cm^{-1} (ν_2), 564.5 cm^{-1} (ν_4), 601.8 cm^{-1} (ν_4), 965.5 cm^{-1} (ν_1), 1033.7 cm^{-1} (ν_3) and 1091.5 cm^{-1} (ν_3). The 1033.7 and 1091.5 cm^{-1} vibrational bands are the triply degenerate ν_3 antisymmetric stretching of the phosphate groups (see **Figure S7**). The 965.5 cm^{-1} band is the non-degenerate ν_1 symmetric stretching of the PO_4^{3-} groups. The 564.5 and 601.8 cm^{-1} peaks are the triply degenerate ν_4 vibration of the O–P–O groups, and the 472.2 cm^{-1} peak is the doubly degenerate ν_2 bending of the O–P–O bongs. The vibrational transition at 3424 cm^{-1} corresponds to a stretching mode (ν(OH)) and those at 630 cm^{-1}

and 3571 cm^{-1} to a vibrational mode (L(OH)) of the OH^- groups, what confirms the presence of hydroxyl groups in the lattice structure [2,3].

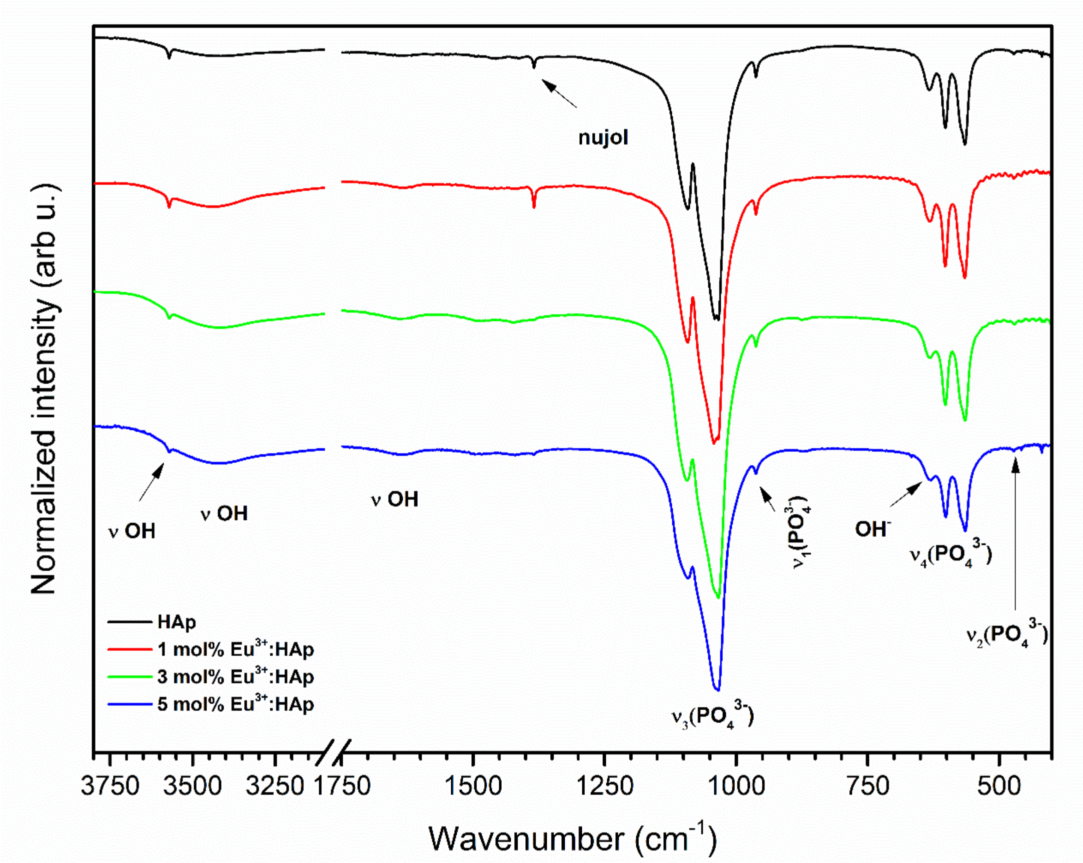

Figure S7. IR spectra of the Eu^{3+}:$Ca_{10}(PO_4)_6(OH)_2$ nanoparticles as a function of optically active ions concentration.

S3.3. Thermal properties

Table S1. The thermal parameters form the first heating DSC curves.

sample	T_g (°C)	T_{cc}^{onset} (°C)	T_{cc} (°C)	ΔH_{cc} (J/g)	$T_{\alpha'-\alpha}$ (°C)	$\Delta H_{\alpha'-\alpha}$ (J/g)	T_m (°C)	ΔH_m (J/g)
PLLA	61.1	91.6	95.6	-34.56	159.2	-7.89	180.9	46.68
PLLA/HAp	59.8	89.0	92.6	-34.86	160.5	-6.57	178.6	46.78
PLLA/ 1 mol%Eu^{3+}:HAp	62.3	84.0	88.7	-22.29	160.8	-6.32	177.2	46.98
PLLA/ 3 mol% Eu^{3+}:HAp	59.4	82.2	87.5	-20.99	159.5	-6.69	177.3	47.60
PLLA/ 5 mol% Eu^{3+}:HAp	61.0	82.6	87.5	-20.78	159.7	-6.66	177.4	47.10

Table S2. The thermal parameters form the cooling DSC curves.

sample	T_c^{onset} (°C)	T_c (°C)	ΔH_c (J/g)	T_g (°C)
PLLA	114.8	107.7	-32.79	67.1
PLLA/HAp	117.1	110.5	-36.34	64.7

PLLA/1 mol% Eu^{3+}:HAp	132.9	125.6	-44.93	62.4
PLLA/3 mol% Eu^{3+}:HAp	133.9	127.6	-45.35	61.7
PLLA/5 mol% Eu^{3+}:HAp	131.9	124.1	-43.33	62.5

Table S3. The thermal parameters form the second heating DSC curves.

sample	T_g (°C)	$T_{\alpha'\text{-}\alpha}$ (°C)	$\Delta H_{\alpha'\text{-}\alpha}$ (J/g)	T_m (°C)	T_{m2} (°C)	ΔH_m (J/g)
PLLA	65.8	164.5	-2.51	179.9	-	42.30
PLLA/HAp	65.1	165.8	-1.06	179.4	-	44.16
PLLA/1 mol% Eu^{3+}:HAp	66.8	-	-	176.1	181.2	51.12
PLLA/3 mol% Eu^{3+}:HAp	62.8	-	-	176.4	181.2	51.39
PLLA/5 mol% Eu^{3+}:HAp	62.8	-	-	175.9	181.2	49.67

S3.4. Spectroscopic properties

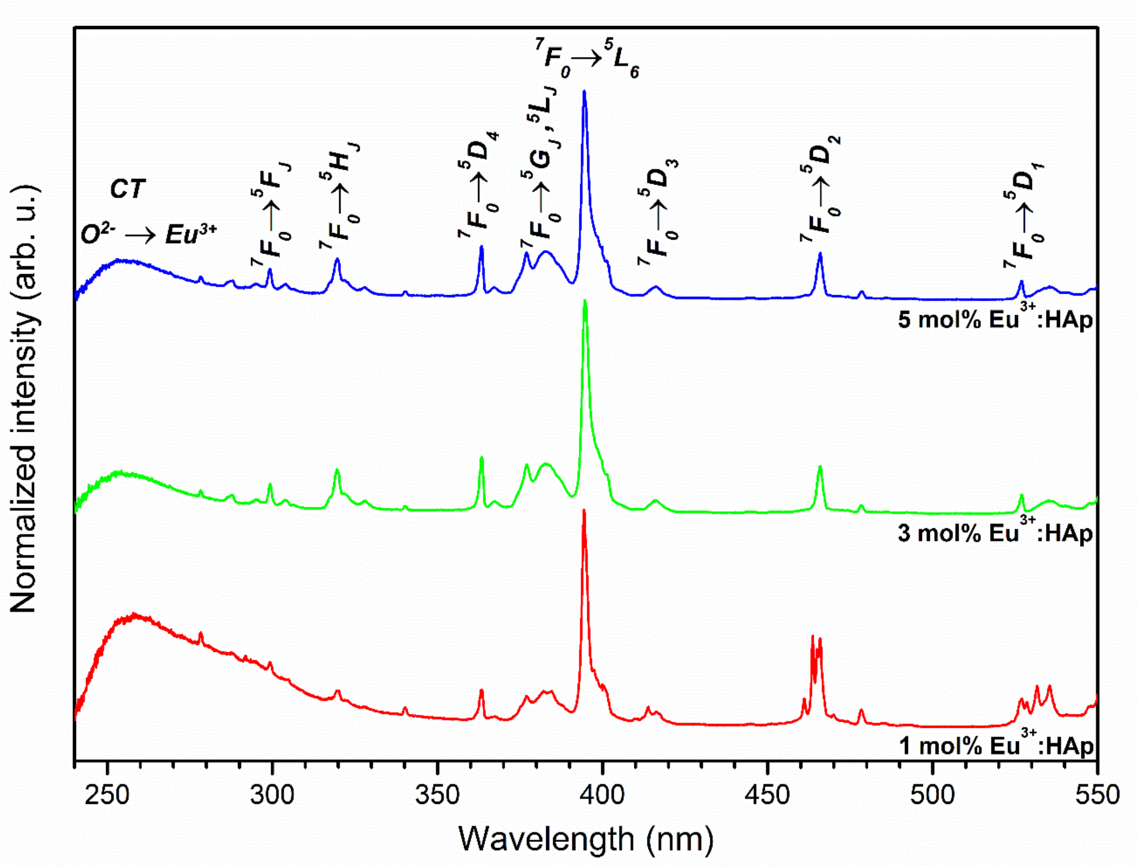

Figure S8. Excitation spectra of the $Ca_{10}(PO_4)_6(OH)_2:Eu^{3+}$ nanoparticles as a function of optically active ions.

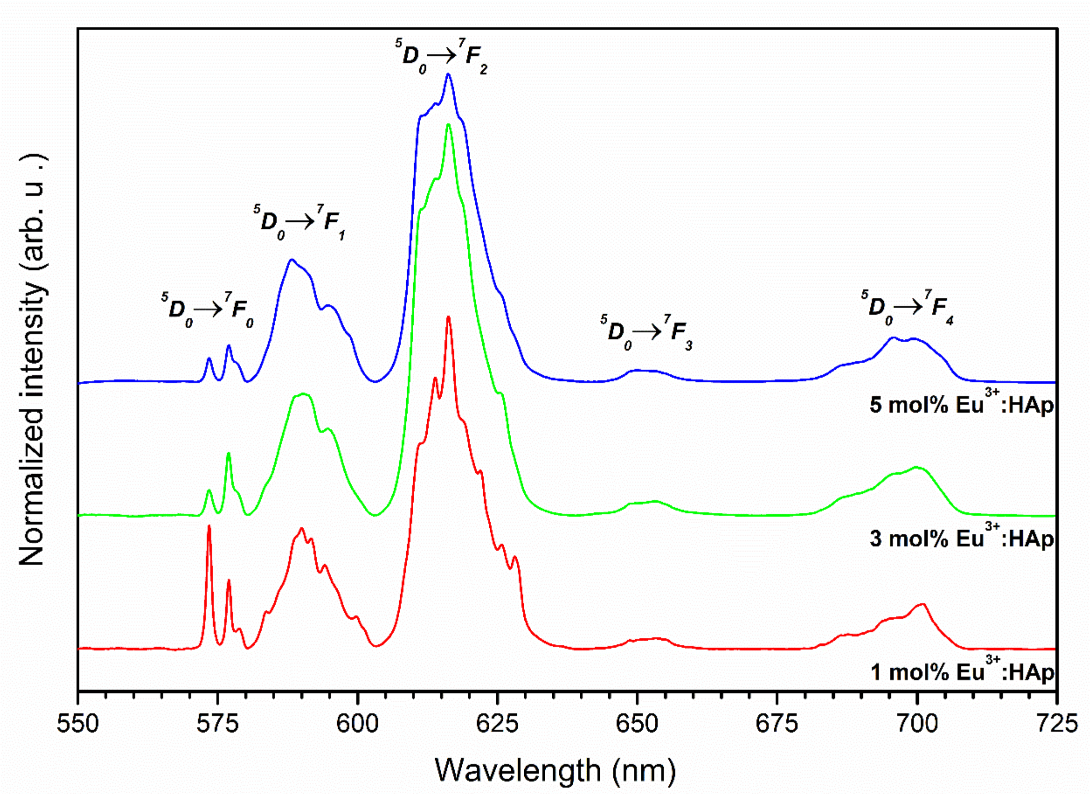

Figure S9. Emission spectra of the $Ca_{10}(PO_4)_6(OH)_2$:Eu^{3+} nanoparticles as a function of optically active ions.

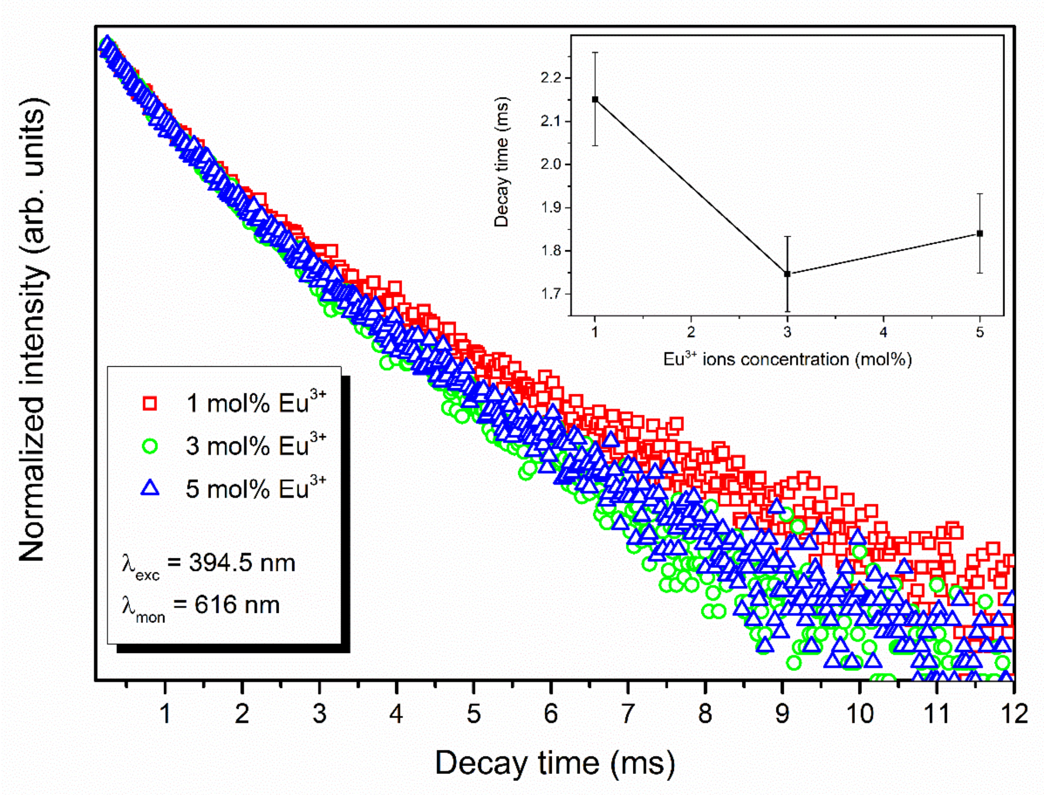

Figure S10. Life times of the $Ca_{10}(PO_4)_6(OH)_2$:Eu^{3+} nanoparticles as a function of optically active ions.

References

1. Balan, E.; Delattre, S.; Roche, D.; Segalen, L.; Morin, G.; Guillaumet, M.; Blanchard, M.; Lazzeri, M.; Brouder, C.; Salje, E.K.H. Line-broadening effects in the powder infrared spectrum of apatite. *Phys. Chem. Miner.* **2011**, *38*, 111–122, doi:10.1007/s00269-010-0388-x.
2. Guerra-López, J.R.; Echeverría, G.A.; Güida, J.A.; Viña, R.; Punte, G. Synthetic hydroxyapatites doped with Zn(II) studied by X-ray diffraction, infrared, Raman and thermal analysis. *J. Phys. Chem. Solids* **2015**, *81*, 57–65, doi:10.1016/j.jpcs.2015.01.017.
3. Yuan, Q.; Wu, J.; Qin, C.; Xu, A.; Zhang, Z.; Lin, S.; Ren, X.; Zhang, P. Spin-coating synthesis and characterization of Zn-doped hydroxyapatite/polylactic acid composite coatings. *Surf. Coatings Technol.* **2016**, *307*, 461–469, doi:10.1016/j.surfcoat.2016.09.021.
